# Day‐to‐day fasting plasma glucose variability on the short‐term prognosis of ST‐segment elevation myocardial infarction: A retrospective cohort study

**DOI:** 10.1002/clc.23899

**Published:** 2022-09-07

**Authors:** Ming Yi, Qing Cao, Wen‐hui Tang, Qiang Liu, Xiao Ke

**Affiliations:** ^1^ Department of Cardiology Liuyang Hospital of Traditional Chinese Medicine Liuyang China; ^2^ Department of Clinical Medicine University of South China Hengyang China; ^3^ Department of Internal Medicine, The Fourth Hospital of Changsha Changsha Hospital Affiliated to Hunan Normal University Changsha China; ^4^ Department of Cardiology, Fuwai Hospital Chinese Academy of Medical Sciences, Shenzhen, (Shenzhen Sun Yat‐sen Cardiovascular Hospital) Shenzhen China; ^5^ Department of Cardiology Shenzhen Traditional Chinese Medicine Hospital Shenzhen China

**Keywords:** fasting plasma glucose, glycemic variability, MACE, STEMI

## Abstract

**Background and Hypothesis:**

Glycemic variability in one fact that explain the differences in cardiovascular outcomes. The short‐term fasting plasma glucose (FPG) variability may have an on major adverse cardiovascular events (MACE) in type 2 diabetes mellitus (T2DM) patients with ST‐segment elevation myocardial infarction (STEMI).

**Methods:**

This study retrospectively analyzed T2DM patients who underwent emergent percutaneous coronary intervention (PCI) due to STEMI in Fuwai Hospital, Chinese Academy of Medical Sciences, Shenzhen, between January 2016 and March 2020. All patients underwent at least 5 FPG measurements during the perioperative period. FPG variability score (FPG‐VS) was defined as the percentage of the number of FPG variations > 1 mmol/L between two adjacent FPG measurements. The Cox proportional‐hazards model was used to estimate the relationship between FPG‐VS and MACE. A validation set was utilized to further evaluate the prognostic value of FPG‐VS in a standardized STEMI diabetic diet cohort following PCI intervention.

**Results:**

A total of 612 patients were included in the retrospective cohort study. In comparison to the minimum quintile, FPG‐VS > 60% was associated with an increased risk of 30‐day MACE. Moreover, compared to FPG‐VS ≤ 20%, the FPG‐VS > 80% group had a higher risk of MACE (odd ratio [OR] = 4.87, 95% confidence interval [CI]: 2.55−5.28), recurrent angina pectoris (OR = 5.43, 95% CI: 2.27−8.27), nonfatal myocardial infarction (OR = 5.00, 95% CI: 2.47−7.69), heart failure (OR = 3.70, 95% CI: 1.92−5.54), malignant arrhythmia (OR = 4.63, 95% CI: 1.12−6.25) and cardiac death (OR = 1.41, 95% CI: 0.17−1.97). Consistent results were obtained after adjustment for HbA1c, demonstrating the robustness of FPGFPG‐VS. Moreover, the standard diet intervention group had a lower FPG‐VS index as well as a lower incidence of MACE.

**Conclusion:**

Higher FPG variability is associated with an increased risk of MACE within 30 days in diabetes patients receiving PCI for STEMI. A standardized diet may improve the prognosis of STEMI patients by reducing the FPG‐VS.

## Key points

• The fasting plasma glucose variability score (FPG‐VS), a new glycemic variability evaluation index, could reflect FPG variability in ST‐segment elevation myocardial infarction (STEMI) patients.
• High FPG‐VS levels were associated with a poor short‐term prognosis in STEMI patients.
• Using FPG‐VS to manage daily glycemic variability might improve STEMI patients' prognosis.

## 1 INTRODUCTION

Type 2 diabetes mellitus (T2DM) is a significant risk factor for cardiovascular disease (CVD), and glycemic control level has been shown to be positively associated with the risk of acute and chronic CVD complications in T2DM patients. A substantial body of evidence suggests that good glycemic control could significantly decrease the incidence of CVD complications in T2DM patients.^1^, ^2^ However, a report by the Action to Control Cardiovascular Risk in Diabetes trial revealed that glycemic control to near normalization could also increase the incidence of cardiovascular events.^3^, ^4^ Growing evidence suggests that lowering glycemic variability is an important factor influencing diabetes‐related cardiovascular risk.^5^, ^6^ Glycemic variability is a component of glycemic control that can be detected using various methods. Indexes for evaluating glycemic variability during hospitalization, including the standard deviation (SD), mean (M) and coefficient of variation (CV) of fasting plasma glucose (FPG), were found to be independent predictors of cardiovascular risk in T2DM patients.^7^, ^8^ A prospective observational study at Ruijin Hospital in Shanghai found that the variability index independent of FPG mean (VIM) was an independent prognostic factor for adverse left ventricular remodeling in T2DM patients with ST‐segment elevation myocardial infarction (STEMI).^9^ Increasing evidence suggests that glycemic variability is an independent risk factor for diabetes complications,^5^, ^10^ since not only can it reflect the effect of glycemic control over the course of the disease, but also predict the risk of hyperglycemia or hypoglycemia. By definition, glycemic variability is quantified based on the glycemic measurements within 2 days or based on HbA1c, or over a longer period.^5^, ^11^

By reviewing the studies domestically and abroad, we found that the prognostic significance of FPG level during hospitalization, the glycemic level at admission, and maximum glycemic level during acute coronary syndrome,^12^, ^13^ as well as the significance of FPG‐CV, FPG‐SD, and VIM in the long‐term prognosis of CVD,^9^, ^10^ have been well documented; however, the short‐term effect remains poorly understood. To that end, our study retrospectively analyzed the clinical data of T2DM patients who underwent emergent percutaneous coronary intervention (PCI) due to STEMI at Fuwai Hospital, Chinese Academy of Medical Sciences, Shenzhen. The FPG level was dynamically monitored, and a new index termed FPG variability score (FPG‐VS) was defined as the frequency of FPG variation >1 mmol/L, in line with the calculation methodology for CV and VIM.^14^ We found that this index could better indicate the direct relationship between glycemic variability and clinical outcome and serve as a new clinical indicator for nonpharmaceutical glycemic variability management. In addition, this study also looked at 30‐day major adverse cardiovascular events (MACE) to provide a reference for clinical management.

Diet control is the basis of diabetes treatment,^15^ and intensive dietary control is an important strategy to reduce glycemic variability.^11^ Therefore we validated the prognostic value of FPG‐VS in a training set, which suggested that standard dietary intervention could reduce the FGP‐VS index level and improve short‐term outcomes in STEMI patients following PCI.

## 2 METHODS

### 2.1 Research subjects

In total, this retrospective case‐control study enrolled 612 T2DM patients who underwent PCI for STEMI between January 2016 and March 2020. All included patients were from a previous study (*n* = 1, 846)^16^ registered with the Fuwai Hospital, Chinese Academy of Medical Sciences, Shenzhen (ChiCTR2100043897). Patients who were followed up signed the Informed Consent or agreed to be followed up by phone.

### 2.2 Testing set

A total of 210 STEMI patients who underwent emergency PCI at Changsha Fourth Hospital were recruited between December 2020 and December 2021. Patients were randomly assigned to a standardized dietary care group or control group. Both groups received basic insulin therapy, routine health education, glucose monitoring, exercise counseling, and other nursing care. The standardized dietary care group received between 1400 and 1800 kcal daily. The ratio of macronutrients provided was 45%–55% carbohydrates, 15%–25% proteins, and 25%–35% fat, in addition to a recommended intake of 20–40 g/day of fiber in the form of vegetables and fruits (ChiCTR2100042809).

### 2.3 Inclusion and exclusion criteria

The inclusion criteria for this study were as follows: (1) Patients who were diagnosed as STEMI conforming to the 2016 American Colleage of Cardiology/American Heart Association guidelines, (2) Patients who underwent emergency PCI, (3) Patients who had FPG measurement ≥5 times during hospitalization, (4) FPGs were determined using the glucose oxidase method. The exclusion criteria were as follows: (1) Patients who had undergone PCI before the study, (2) Patients who had FPG measurement <5 times during hospitalization, (3) Patients who underwent coronary artery bypass grafting or bridging stent placement during hospitalization, (4) Patients who experienced diabetic ketoacidosis; (5) Patients with estimated survival <30 days.

### 2.4 Data collection

Patient basic information included demographic data, medical history, history of diabetes medication, coronary artery angiography manifestation, and type of PCI intervention. MACE included recurrent angina pectoris (AP), nonfatal myocardial infarction (MI) (including PCI‐related MI), malignant arrhythmia, heart failure (HF), and cardiac death. PCI‐related MI was diagnosed using cTn increase >fivefold. Malignant arrhythmias included sinus arrest, type 2 second‐degree atrioventricular (AV) block, third‐degree AV block, > LOWN4 ventricular premature, ventricular tachycardia, and ventricular fibrillation.

### 2.5 FPG‐VS definition

**Figure 1:**
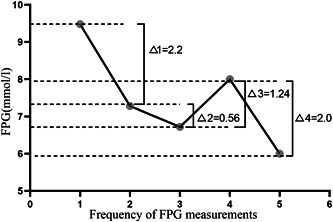
Calculation of the FPG‐VS index. FPG‐VS = number of FPG variation >1 mmol/L between two adjacent measurements/(total number of FPG measurement during hospitalization −1) × 100%. Taking a patient with 5 FPG measurements as an example, Δ1, Δ3, and Δ4 > 1 mmol/L, then, FPG‐VS = 3/(5−1) × 100% = 75%. FPG‐VS, fasting plasma glucose variability score

FPG levels during hospitalization were recorded. FPG‐VS = number of FPG variation >1 mmol/L between two adjacent measurements/(total number of FPG measurement during hospitalization −1) × 100% (Figure 1).

### 2.6 Ethical issues

The study conformed to the principles outlined in the Declaration of Helsinki,^17^ and all participants gave written informed consent and were registered on ChineseClinicaltrial.gov (ChiCTR2100042809 and ChiCTR2100043897).

### 2.7 Statistical analysis

IBM SPSS 25.0 was utilized for statistical analysis. Measurement data expressed as $\bar{\chi }\pm S$ were compared using a two‐sample *t*‐test, one‐way analysis of variance, or Mann−Whitney *U* test. Enumeration data expressed as *n* (%) were compared using the *χ* ^2^ test. The Cox proportional‐hazards model was used to estimate the relationship between FPG‐VS and each outcome. After adjusting for age, gender and HbA1c, the relationship between each outcome and FPG‐VS categories (0%−20%, 20%−40%, 40%−60%, 60%−80%, and >80%) was further explored following *α* = .05.

## 3 RESULTS

### 3.1 Baseline characteristics

**Figure 2:**
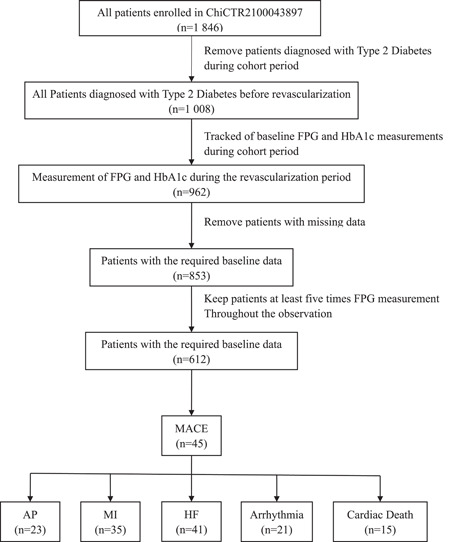
Study workflow of the included patients. AP, Angina Pectoris; FPG, Fasting Plasma Glucose; HbA1c, Glycated Hemoglobin; HF, Heart Failure; MACE, Major Adverse Cardiovascular Events; MI, Myocardial Infarction.

As shown in Figure 2, we included 612 patients consisting of 487 (80.6%) male and 117 (19.4%) female patients. The mean age was 56.3 ± 11.1 (years), baseline FPG level was 8.8 ± 3.4 (mmol/L), and FPG was measured throughout the study duration at different timepoints. The median number of visits was 6 (IQR: 5−16). The difference in baseline data under different FPG‐VS categories (0%−20%, 20%−40%, 40%−60%, 60%−80%, and >80%) is shown in Table 1. As expected, high FPG‐VS was accompanied by a lower insulin intensive therapy. The insulin treatment rate of patients with FPG‐VS > 80% was only 37.5% (9/24, *p* < .05).

**Table 1:** Difference in baseline data across FPG‐VS categories

	FPG‐VS Categories				
	≥0−≤20	>20~≤40	>40−≤60	>60−≤80	>80−≤100
*n*	234	145	123	86	24
Age(years)	54.6 ± 9.6	59.0 ± 13.7	53.2 ± 8.8	59.9 ± 11.6	60.4 ± 6.2
Male Sex, *n* (%)	193 (82.5%)	111 (81.0%)	105 (85.4%)	62 (72.1%)	16 (66.8%)
Hypertension, *n* (%)	84 (35.8%)	47 (32.4%)	77 (62.6%)	58 (67.4%)	8 (33.3%)
Dyslipidemia, *n* (%)	155 (66.2%)	78 (53.8%)	94 (76.4%)	58 (67.4%)	14 (58.3%)
eGFR < 60 ml. min^−1^.1.73 m^−2^, *n* (%)	3 (0.4%)	1 (0.7%)	3 (2.4%)	1 (1.2%)	1 (4.1%)
Diabetes diagnosis (years)	2.46 ± 0.6	2.61 ± 1.1	2.81 ± 1.0	2.50 ± 1.2	2.91 ± 0.4
Ever smokingn, *n* (%)	81 (34.6%)	69 (47.6%)	85 (69.1%)	45 (52.3%)	12 (50.0%)
Ever regular alcohol, *n* (%)	19 (3.1%)	8 (5.5%)	1 (0.8%)	6 (0.7%)	3 (12.5%)
BMI (kg/m^2^)	24.0 ± 0.6	24.4 ± 1.3	24.2 ± 1.5	24.3 ± 1.7	24.3 ± 1.4
Heart rate (bpm)	70.3 ± 8.7	82.5 ± 15.1	77.1 ± 1.2	73.3 ± 1.1	72.7 ± 1.6
Systolic blood pressure (mmHg)	129.1 ± 1.3	123.8 ± 1.8	134.7 ± 2.4	138 ± 2.1	147.7 ± 4.2
Diastolic blood pressure (mmHg)	76.0 ± 0.8	73.9 ± 1.0	81.8 ± 1.7	80.9 ± 1.0	84.4 ± 1.9
Baseline at FPG (mmol/L)	7.16 ± 0.9	7.65 ± 1.8	8.76 ± 2.4	8.28 ± 3.3	9.98 ± 4.1
Baseline at HbA1C (%)	6.6 ± 0.7	7.3 ± 1.5	7.1 ± 1.5	6.6 ± 1.5	7.6 ± 1.8
LDL (mmol/L)	3.04 ± 1.2	3.03 ± 1.2	3.06 ± 1.2	3.12 ± 1.1	3.43 ± 1.1
HDL (mmol/L)	1.17 ± 0.4	1.04 ± 0.2	1.02 ± 0.3	1.11 ± 0.2	1.09 ± 0.2
TG (mmol/L)	2.64 ± 5.0	2.08 ± 1.89	2.17 ± 1.5	2.07 ± 1.1	2.21 ± 1.6
Drugs treatment					
ACEi	158 (67.5%)	103 (71.1%)	87 (70.1%)	48 (55.8%)	8 (33.3%)
ARB			18 (14.6%)	26 (30.2%)	7 (29.2%)
β receptor inhibitor, *n* (%)	197 (84.2%)	129 (89.0%)	104 (84.6%)	69 (80.2%)	24 (100%)
Statin, *n* (%)	234 (100%)	145 (100%)	123 (100%)	79 (91.9%)	24 (100%)
CCB, *n* (%)	4 (1.7%)	10 (6.9%)	38 (30.9%)	30 (34.9%)	3 (12.5%)
Sulfonylurea, *n* (%)	1 (0.4%)	17 (11.7%)	11 (8.9%)	23 (26.7%)	4 (16.7%)
Metformin, *n* (%)	40 (17.1%)	9 (6.2%)	22 (17.9%)	41 (47.7%)	11 (45.8%)
α‐glucosidase inhibitor, *n* (%)	39 (16.7%)	19 (13.1%)	19 (15.4%)	44 (51.2%)	5 (20.8%)
Insulin, *n* (%)	77 (32.9%)	35 (24.1%)	37 (30.1%)	2 (2.3%)	4 (16.7%)
Length of stay (days)	6.7 ± 0.8	7.2 ± 1.4	7.1 ± 1.4	8.2 ± 4.0	8.7 ± 4.4

Abbreviations: ACEi, Angiotensin‐converting‐enzyme inhibitors; ARB, angiotensin ii receptor blockers; BMI, body mass index; CCB, calcium channel blocker; eGFR, estimated glomerular filtration rate; FPG, fasting plasma glucose; HDL, high‐density lipoprotein; LDL, low‐density lipoprotein; TG: triglyceride.

As shown in Supporting Information: Figure 1, we ultimately included 210 patients in the testing set, and Supporting Information: Table 1 summarizes the baseline characteristics of study participants. Overall, the mean age of the study population was 59.0 ± 1.2 years, and 85% were male subjects (*n* = 85). In terms of laboratory findings at admission, there were no statistically significant differences between the two study groups with respect to baseline sociodemographic characteristics and other cardiovascular risk factors. Baseline levels of HbA1c (*p* = .780) and FPG (*p* = .050) between the intervention and control groups were not statistically different, and Supporting Information: Table 1 shows the specific details of the baseline characteristics of the two groups.

### 3.2 High FPG‐VS predicts a high risk of MACE

**Figure 3:**
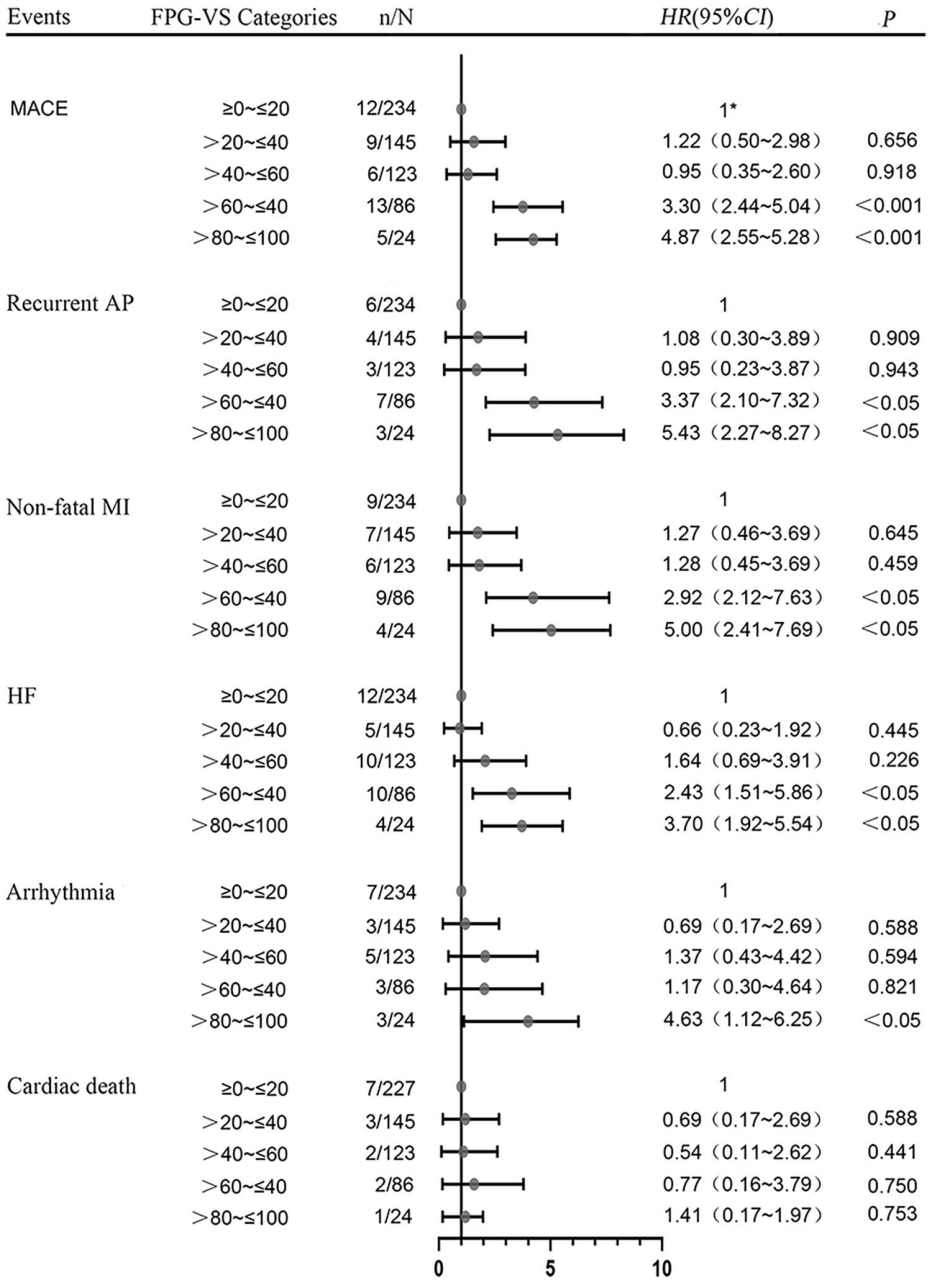
Relationship between FPG‐VS and each clinical outcome. AP, angina pectoris; HR, hazard ratio; MACE, major adverse cardiovascular events; MI, myocardial infarction; HF, heart failure. *As a reference.

Cox proportional‐hazards analysis revealed that FPG‐VS > 60% was associated with an increased risk for all outcomes except cardiac death when compared to the reference FPG‐VS (0%−20%). For instance, FPG‐VS ranging 80%−100% was associated with an increased risk of MACE (OR = 4.87, 95% CI: 2.55−5.28, *p* < .001), recurrent AP (OR = 5.43, 95% CI: 2.27−8.27, *p* < .05), nonfatal MI (OR = 5.00, 95% CI: 2.47−7.69, *p* < .05), HF (OR = 3.70, 95% CI: 1.92−5.54, *p* < .05) and malignant arrhythmia (OR = 4.63, 95% CI: 1.12−6.25, *p* < .05). No association with cardiac death (OR = 1.41, 95% CI: 0.17−1.97, *p* = .753) was found (Figure 3).

### 3.3 Subgroup analysis

**Figure 4:**
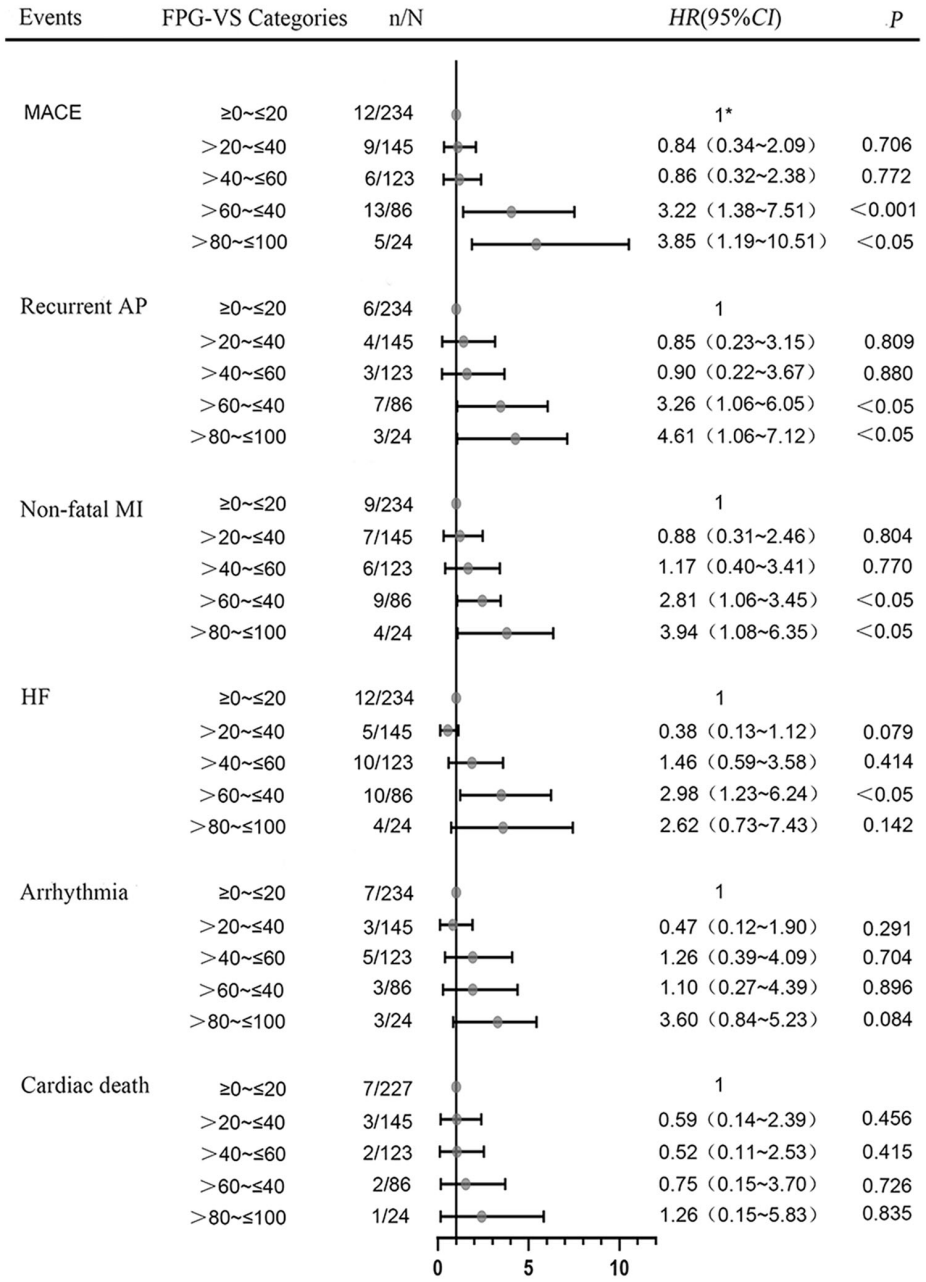
Relationship between FPG‐VS and each clinical outcome after adjustment for HbA1c. AP, Angina Pectoris; HF, Heart Failure; HR, hazard ratio, MACE, Major Adverse Cardiovascular Events; MI, Myocardial Infarction. *As a reference.

Since both FPG‐VS and HbA1c are indicators for glycemic variability evaluation, the HbA1c level (from the beginning of PCI to complication incidence) was adjusted, resulting in similar results in the majority of outcomes except for HF, malignant arrhythmia, and cardiac death (Figure 4). After adjustment for age, gender, baseline body mass index, and time of insulin use, FPG‐VS still exhibited significant associations with recurrent AP, nonfatal MI, HF, and malignant arrhythmia (Supporting Information: Figure S2). No significant differences were observed in other subgroup analyses of FPG‐M (Supporting Information: Figure S3), FPG‐SD (Supporting Information: Figure S4), and FPG‐CV (Supporting Information: Figure S5).

### 3.4 Testing set analysis

Consistent with the above findings, analysis of the testing cohort revealed a close association between hyperglycemia variability and the short‐term prognosis of STEMI patients, indicating that FPG‐VS is a robust indicator for evaluating glycemic variability.

Notably, the glycemic variability in the diet intervention group was lower, and the FPG‐VS index was significantly lower compared to the control group (0.60 vs. 0.34, *p* < .0001), as shown in Supporting Information: Figure 6A. Additionally, it was accompanied by a shorter hospital stay (7.89 vs. 6.44, *p* < .001), as detailed in Supporting Information: Figure 6B/C. Furthermore, the incidence of MACE during hospitalization in the intervention group was significantly lower than in the control group (*p* < .001), as shown in Supporting Information: Figure 6D.

## 4 DISCUSSION

There is currently no recognized gold standard for quantifying glycemic variability. Herein, to assess FPG variability, we introduced a new index, FPG‐VS, for glycemic variability evaluation based on FPG levels during the perioperative period of T2DM patients receiving PCI for STEMI. Our retrospective case‐control study found that high FPG‐VS was closely associated with the short‐term prognosis of STEMI patients, indicating that glycemic variability could predict the short‐term risk of CVDs in T2DM patients. Importantly, the testing set revealed that diet management might improve the prognosis of STEMI patients by lowering the FPG‐VS index level.

### 4.1 Effect of FPG‐VS on short‐term prognosis

Coronary lesions in diabetes patients generally have poor clinical outcomes, and poor glycemic control further results in an increased risk of MACE.^10^, ^18^ On the other hand, good perioperative glycemic control (HbA1c ≤ 7.0%) is associated with a significant reduction in the incidence of in‐stent restenosis.^9^ In addition, several previous studies revealed that good glycemic control during the perioperative period in patients undergoing PCI is vital for preventing adverse cardiovascular events postoperatively. However, the effect of perioperative glycemic control remains controversial since the glycemic control in these studies was achieved based on the HbA1c level at a certain time point, without further considering glycemic variability. Indeed, glycemic variability has been reported to be effective in interpreting the incidence of adverse cardiovascular events.^6^ In the present study, we proved that FPG‐VS was an independent risk factor for CVD, consistent with the existing literature.^7^, ^9^, ^13^, ^18^

Most related studies focused on the long‐term impact of glycemic variability on adverse cardiovascular events in diabetes patients,^7^, ^9^, ^14^ while few studies investigated the incidence of MACE during hospitalization. Moreover, we found that short‐term glycemic variability was associated with CVD risk. The results indicated that patients with higher FPG‐VS were at a higher risk of MACE, in agreement with a previous clinical case‐control trial^19^ and an observational study.^9^ Taken together, our findings suggest that high FPG‐VS is a significant independent risk factor for the poor prognosis of diabetes patients, while patients with more stable FPG are more likely to have better clinical outcomes. Moreover, high FPG‐VS during the perioperative period of patients undergoing PCI was also associated with a prolonged hospitalization duration. Nevertheless, these results should be interpreted with caution as we cannot account for the intensification of glucose‐lowering therapy during hospitalization.

Diabetes is a lifelong chronic disease. A proprietary recipe for patients based on the Resident Dietary Guidelines and a low‐calorie dietary treatment to reduce glycemic fluctuations could reduce acute and chronic comorbidities associated with diabetes and ultimately improve biomedical, behavioral, and psychosocial outcomes.^20^ In addition, a previous study revealed the importance of glycemic control during hospitalization to prevent subsequent adverse cardiovascular outcomes in STEMI patients^11^ The present study evaluated FPG variability using the FPG‐VS index, showing that the incidence of MACE in the intervention group was significantly lower compared to the control group, indicating that standardized dietary care intervention on a daily level can effectively reduce the FPG‐VS index. However, reduction of glycemic variation may be achieved by multiple methods, and our findings used day‐to‐day dietary management to confirm the robustness of FPG‐VS's predictive value rather than directly evaluating the efficacy of dietary intervention itself.

### 4.2 Potential mechanism

It is currently unknown how FPG variation affects the short‐term prognosis of diabetic patients undergoing emergent PCI. We surmise that the nonstandard insulin therapy that results in FPG fluctuation might be associated with the short‐term poor prognosis of diabetes patients.^21^ In addition, our results revealed that the rate of insulin use varied significantly across FPG‐VS categories (*p* < .05). For instance, the rates of insulin use were 5.8% and 33.3% in the FPG‐VS ≥ 80% and <20% subgroups, respectively. Moreover, we noted that some patients experienced recurrent episodes of hypoglycemia in the FPG‐VS ≥ 80% subgroup. Previous research has shown that hyperglycemia and glycemic fluctuation can cause endothelial dysfunction by directly or indirectly stimulating the hyperproduction of reactive oxygen species and the formation of advanced glycation end products, which we believe are important basic mechanisms that can induce short‐term adverse events following PCI.^22^

### 4.3 Limitation

Nevertheless, the findings of our study should be interpreted within the context of the following limitations: this was a single‐center retrospective study that analyzed the significance of FPG‐VS for short‐term prognosis in diabetes patients receiving PCI. Therefore, more in‐depth investigations are warranted to further validate our results.

## 5 CONCLUSION

High levels of FPG‐VS were independently associated with the increased risk of MACE in T2DM patients that underwent emergent PCI for STEMI.

## AUTHOR CONTRIBUTIONS

Xiao Ke and Qiang Liu conceived the project. Ming Yi and Qing Cao were responsible for the experimental design and application and writing the manuscript. Ming Yi and Wen‐hui Tangperformed data analysis for the manuscript. All authors read and approved the final manuscript.

## CONFLICT OF INTEREST

The authors declare no conflict of interest.

## Supporting information


**Figure S1**. The workflow of the patients' randomized recruitment process. The study workflow. *This flowchart displays the selection process of the participants included in the study. There were no losses to follow‐up or study arm crossovers during the 30 days of study follow‐up.

**Figure S2. Relationship between FPG‐VS and each clinical outcome after adjustment for multiple factors**. HR, hazard ratio, MACE: Major Adverse Cardiovascular Events; AP: Angina Pectoris; MI: Myocardial Infarction; HF: Heart Failure. *: as a reference.

**Figure S3. Relationship between FPG‐M and each clinical outcome**. FPG‐M: mean of FPG, HR, hazard ratio, MACE: Major Adverse Cardiovascular Events; AP: Angina Pectoris; MI: Myocardial Infarction; HF: Heart Failure. *: as a reference.

**Figure S4. Relationship between FPG‐SD and each clinical outcome**. FPG‐SD: standard deviation of FPG, HR, hazard ratio, MACE: Major Adverse Cardiovascular Events; AP: Angina Pectoris; MI: Myocardial Infarction; HF: Heart Failure. *: as a reference.

**Figure S5. Relationship between FPG‐CV and each clinical outcome**. FPG‐CV: coefficient of variation of FPG, HR, hazard ratio, MACE: Major Adverse Cardiovascular Events; AP: Angina Pectoris; MI: Myocardial Infarction; HF: Heart Failure. *: as a reference.

**Figure S6. Standardized dietary care reduces FPV variability and improves short‐term outcomes in STEMI patients**. (**A**) The FPG‐VS levels in the control and intervention groups. (**B/C**) The length of stay in the control and intervention group and its relationship with MACE independently. (**D**) Occurrence of MACE in the control and intervention groups. *P＜0.05，**P＜0.01，***P＜0.01.

**Supplement** Table 1. Baseline characteristics of the random control study population. eGFR: estimated glomerular filtration rate, BMI: body mass index, LDL: low‐density lipoprotein, HDL: high‐density lipoprotein, TG: triglyceride, ACEi: Angiotensin‐converting‐enzyme inhibitors, ARB: angiotensin II Receptor Blockers, CCB: calcium channel blocker.

## Data Availability

The original contributions presented in the study are included in the article/Supporting Information Material, further inquiries can be directed to the corresponding authors.
