# Supplementary material for: Day‐to‐day fasting plasma glucose variability on the short‐term prognosis of ST‐segment elevation myocardial infarction: A retrospective cohort study
Source: Clin Cardiol. 2022 Sep 7;45(12):1246–54. doi: 10.1002/clc.23899 (PMC9748763; doi:10.1002/clc.23899)
Supplement: Supplementary file 1 — Figure S1. The workflow of the patients' randomized recruitment process. The study workflow. *This flowchart displays the selection process of the participants included in the study. There were no losses to follow‐up or study arm crossovers during the 30 days of study follow‐up. [file CLC-45-1246-s001.pdf]

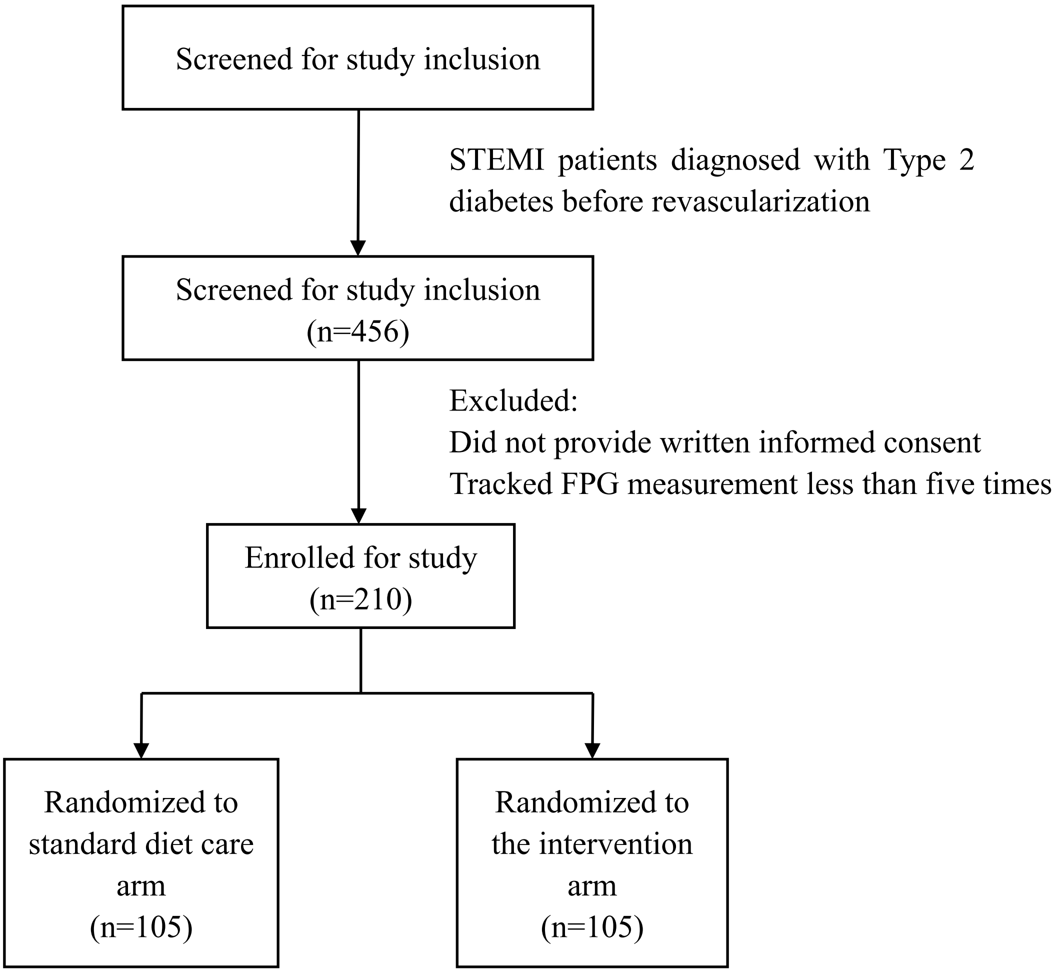

The flow of the study participants. This flowchart displays the flow of the participants included in the study. There were no losses to follow-up or study arm crossovers during the 30 days study follow-up
