# Supplementary figures and images for: Day‐to‐day fasting plasma glucose variability on the short‐term prognosis of ST‐segment elevation myocardial infarction: A retrospective cohort study
Source: Clin Cardiol. 2022 Sep 7;45(12):1246–54. doi: 10.1002/clc.23899 (PMC9748763; doi:10.1002/clc.23899)

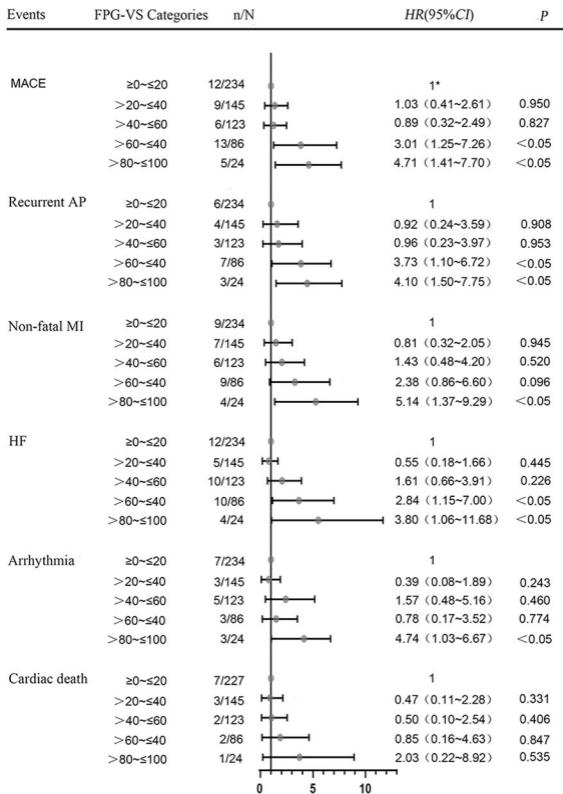

Supplement: Supplementary file 2 — Figure S2. Relationship between FPG‐VS and each clinical outcome after adjustment for multiple factors. HR, hazard ratio, MACE: Major Adverse Cardiovascular Events; AP: Angina Pectoris; MI: Myocardial Infarction; HF: Heart Failure. *: as a reference. [file CLC-45-1246-s003.pdf]

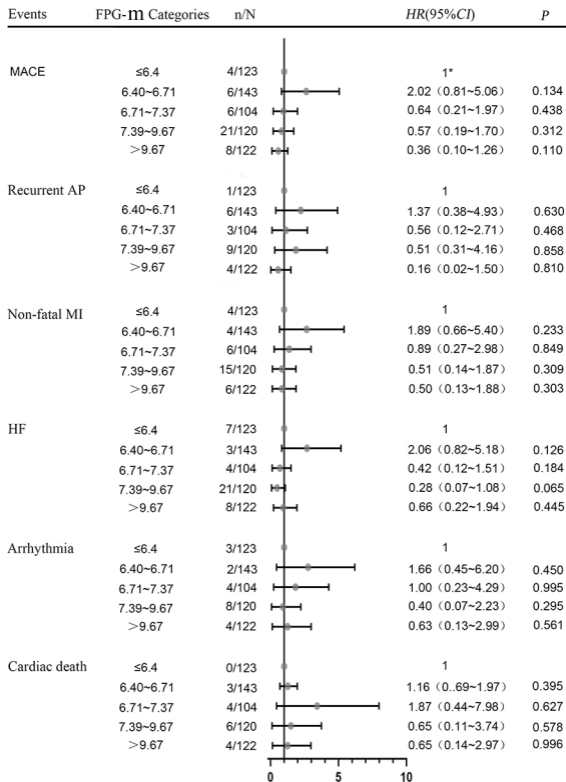

Supplement: Supplementary file 3 — Figure S3. Relationship between FPG‐M and each clinical outcome. FPG‐M: mean of FPG, HR, hazard ratio, MACE: Major Adverse Cardiovascular Events; AP: Angina Pectoris; MI: Myocardial Infarction; HF: Heart Failure. *: as a reference. [file CLC-45-1246-s005.pdf]

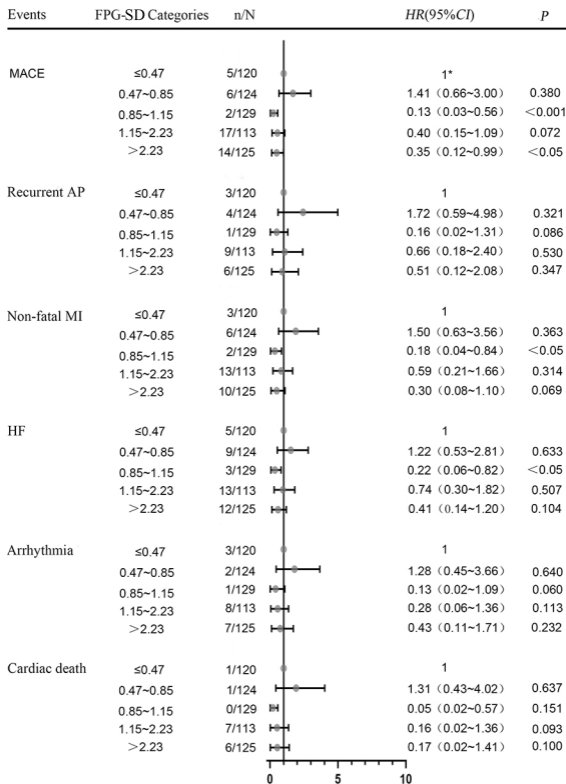

Supplement: Supplementary file 4 — Figure S4. Relationship between FPG‐SD and each clinical outcome. FPG‐SD: standard deviation of FPG, HR, hazard ratio, MACE: Major Adverse Cardiovascular Events; AP: Angina Pectoris; MI: Myocardial Infarction; HF: Heart Failure. *: as a reference. [file CLC-45-1246-s004.pdf]

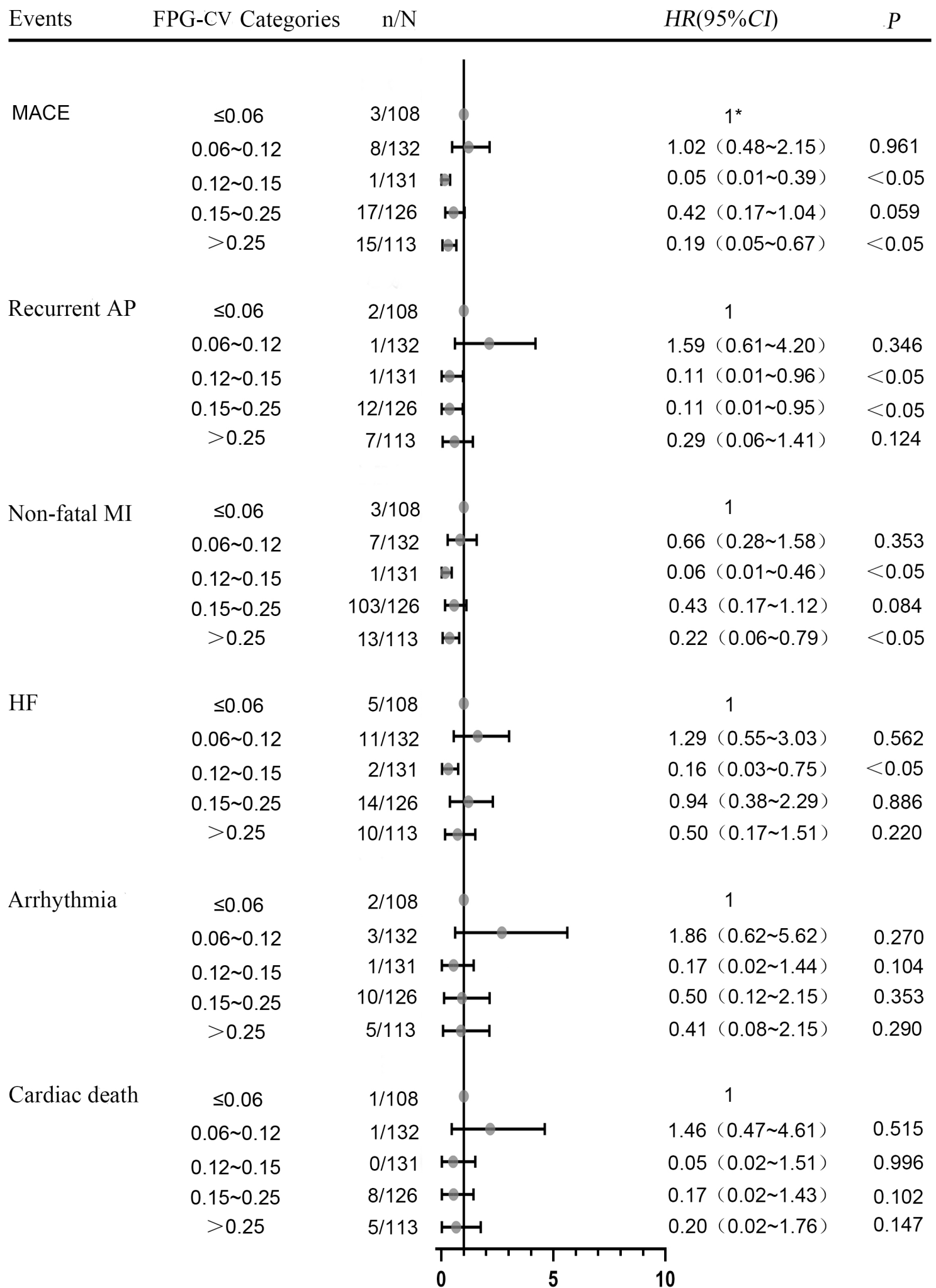

Supplement: Supplementary file 5 — Figure S5. Relationship between FPG‐CV and each clinical outcome. FPG‐CV: coefficient of variation of FPG, HR, hazard ratio, MACE: Major Adverse Cardiovascular Events; AP: Angina Pectoris; MI: Myocardial Infarction; HF: Heart Failure. *: as a reference. [file CLC-45-1246-s002.pdf]

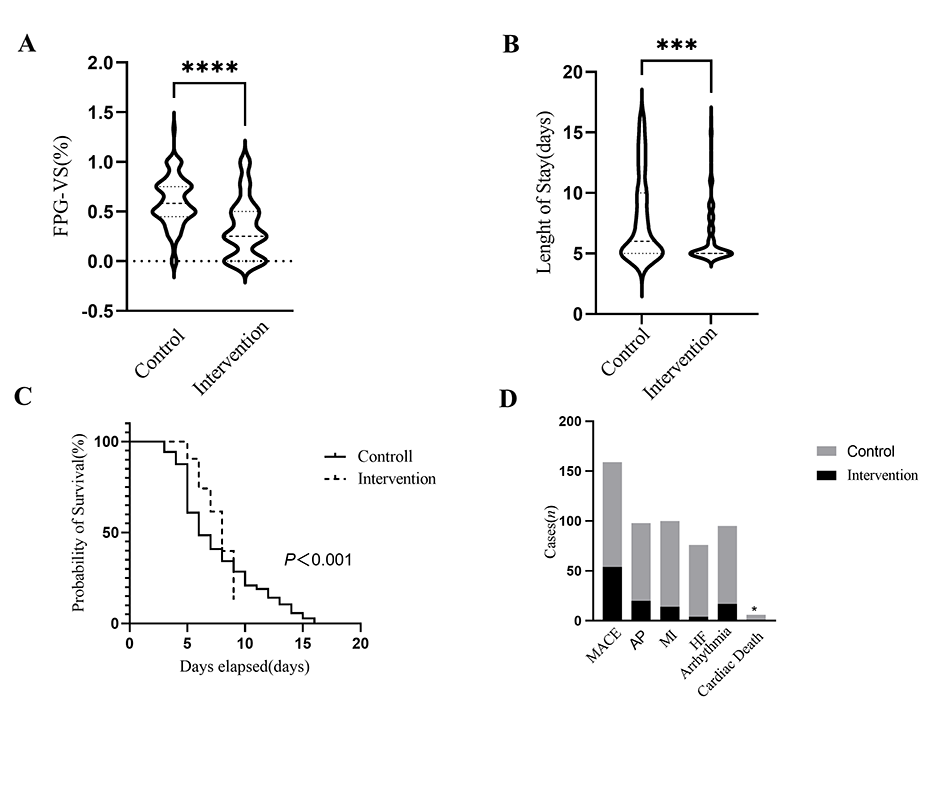

Supplement: Supplementary file 6 — Figure S6. Standardized dietary care reduces FPV variability and improves short‐term outcomes in STEMI patients. (A) The FPG‐VS levels in the control and intervention groups. (B/C) The length of stay in the control and intervention group and its relationship with MACE independently. (D) Occurrence of MACE in the control and intervention groups. *P＜0.05，**P＜0.01，***P＜0.01. [file CLC-45-1246-s007.tif]
