# Supplementary material for: Day‐to‐day fasting plasma glucose variability on the short‐term prognosis of ST‐segment elevation myocardial infarction: A retrospective cohort study
Source: Clin Cardiol. 2022 Sep 7;45(12):1246–54. doi: 10.1002/clc.23899 (PMC9748763; doi:10.1002/clc.23899)
Supplement: Supplementary file 7 — Supplement Table 1. Baseline characteristics of the random control study population. eGFR: estimated glomerular filtration rate, BMI: body mass index, LDL: low‐density lipoprotein, HDL: high‐density lipoprotein, TG: triglyceride, ACEi: Angiotensin‐converting‐enzyme inhibitors, ARB: angiotensin II Receptor Blockers, CCB: calcium channel blocker. [file CLC-45-1246-s006.pdf]

Characteristics	Controll	Intervention	*p*
Age（years)	61.7±1.2	59.0±1.2	0.742
Male Sex,*n* (%）	81（77.1）	85（81.7）	0.412
Hypertension,n(%)	69（65.7）	54（51.4）	0.050
Dyslipidemia,n(%)	62（59.0）	60（57.1）	0.780
eGFR＜60ml.min^{-1}.1.73m^{-2},n(%)	8（7.6）	1(0.9)	0.012
Year of Diabetes diagnosis（years）	3.0±1.1	3.3±1.1	0.495
Ever Smokingn,n(%)	47（44.8）	55（52.4）	0.269
Ever regular Alcohol,n(%)	5（4.8）	5（4.8）	_
BMI(kg/m^2)	24.3±1.5	24.2±1.7	0.650
Heart rate （bpm）	70.3±8.7	77.1±1.2	0.334
Systolic blood pressure（mmHg)	129.1±1.3	134.7±2.4	0.270
Diastolic blood pressure（mmHg)	76.0±0.8	81.8±1.7	0.934
Baseline at FPG（mmol/L）	9.4±3.0	8.9±3.2	0.050
Baseline at HbA1C（%）	7.3±1.8	7.3±1.6	0.780
LDL（mmol/L）	3.04±1.2	3.06±1.2	0.238
HDL（mmol/L）	1.17±0.39	1.02±0.32	0.317
TG（mmol/L）	2.64±4.97	2.17±1.46	0.391
Drugs treatment			
ACEi	72（68.6）	71（67.6）	0.882
ARB	8（7.6）	10（9.5）	0.622
β receptor inhibitor,n(%)	97（92.4）	91（86.7）	0.176
Statin,n(%)	103(100)	103(100)	_
CCB,n(%)	18(17.1)	15(14.3)	0.569
Sulfonylurea,n(%)	14(13.3)	15(14.3)	0.841
Metformin,n(%)	37（35.2）	31（29.5）	0.376
α-glucosidase inhibitor,n(%)	38（36.2）	22（21.0）	0.015
Insulin,n(%)	105(100)	105（100）	_
Length of stay(days)	7.89±3.5	6.44±2.5	＜0.001
